# Utility of Stimulated Thyroglobulin in Reclassifying Low Risk Thyroid Cancer Patients’ Following Thyroidectomy and Radioactive Iodine Ablation: A 7-Year Prospective Trial

**DOI:** 10.3389/fendo.2020.603432

**Published:** 2021-02-24

**Authors:** Anwar A. Jammah, Afshan Masood, Layan A. Akkielah, Shaimaa Alhaddad, Maath A. Alhaddad, Mariam Alharbi, Abdullah Alguwaihes, Saad Alzahrani

**Affiliations:** ^1^ Department of Medicine, King Saud University, Riyadh, Saudi Arabia; ^2^ Obesity Research Center, King Saud University, Riyadh, Saudi Arabia; ^3^ Department of Medicine, Endocrinology Division, Ministry of Health, Kuwait City, Kuwait; ^4^ Faculty of Allied Health Sciences, Kuwait University, Kuwait City, Kuwait; ^5^ Endocrine and Internal Medicine Department, Qassim University, Buraydah, Saudi Arabia; ^6^ Obesity, Endocrine, and Metabolism Center, King Fahad Medical City, Riyadh, Saudi Arabia

**Keywords:** highly sensitive thyroglobulin, differentiated thyroid cancer, stimulated thyroglobulin, reclassification, dynamic risk assessment, DTC recurrence, non-stimulated thyroglobulin

## Abstract

**Context:**

Following total thyroidectomy and radioactive iodine (RAI) ablation, serum thyroglobulin levels should be undetectable to assure that patients are excellent responders and at very low risk of recurrence.

**Objective:**

To assess the utility of stimulated (sTg) and non-stimulated (nsTg) thyroglobulin levels in prediction of patients outcomes with differentiated thyroid cancer (DTC) following total thyroidectomy and RAI ablation.

**Method:**

A prospective observational study conducted at a University Hospital in Saudi Arabia. Patients diagnosed with differentiated thyroid cancer and were post total thyroidectomy and RAI ablation. Thyroglobulin levels (nsTg and sTg) were estimated 3–6 months post-RAI. Patients with nsTg <2 ng/ml were stratified based on their levels and were followed-up for 5 years and clinical responses were measured.

**Results:**

Of 196 patients, nsTg levels were <0.1 ng/ml in 122 (62%) patients and 0.1–2.0 ng/ml in 74 (38%). Of 122 patients with nsTg <0.1 ng/ml, 120 (98%) had sTg levels <1 ng/ml, with no structural or functional disease. sTg levels >1 occurred in 26 (35%) of patients with nsTg 0.1–2.0 ng/ml, 11 (15%) had structural incomplete response. None of the patients with sTg levels <1 ng/ml developed structural or functional disease over the follow-up period.

**Conclusion:**

Suppressed thyroglobulin (nsTg < 0.1 ng/ml) indicates a very low risk of recurrence that does not require stimulation. Stimulated thyroglobulin is beneficial with nsTg 0.1–2 ng/ml for re-classifying patients and estimating their risk for incomplete responses over a 7 years follow-up period.

## Introduction

With marked increase in the incidence rates, thyroid cancer has been deemed to be the most rapidly growing endocrine cancer with an annual increase of 3% (1). This increasing tendency, in the rate of thyroid cancer, has also been reported in Saudi Arabia. The annual Saudi tumor registry report in 2012 placed the prevalence of thyroid cancer at 9.9% with a male/female ratio of 0.3:1 (2–4). Patients with differentiated thyroid cancer with differentiated thyroid cancer (DTC) are commonly diagnosed with papillary thyroid cancer (PTC) (85–90%), followed by follicular thyroid cancer (FTC) (15–10%), then Hurthle thyroid cancer (1–3%), and medullary thyroid cancer (MTC) (3–5%) (5). Although the thyroid tumors have a good prognosis, the rate of recurrence still remains high and consequential at 10–20% (6). In several instances, these recurrences are detected within the first 6 years post thyroidectomy; however, some appear decades later (6).

Currently, the standard initial treatment for patients with high-risk pathology and some with intermediate American Thyroid Association (ATA) is total thyroidectomy, followed by radioactive iodine I-131 remnant ablation (RAI) (7).

Following total thyroidectomy and RAI ablation, serum Tg levels should be undetectable to assure patients that they are excellent responders and at very low risk of recurrence which is observed in majority of them (8). According to the European and American Thyroid Association guidelines, patients with DTC can be considered free of disease when there is no clinical evidence of the tumor, no evidence of the tumor on imaging, and negative serum thyroglobulin (Tg) level. The Tg levels should be undetectable during TSH suppression therapy, i.e. non-stimulated Tg (nsTg) and/or following stimulation (sTg) measurement (9–12). Because of its high cellular specificity, any change in Tg levels serve as a sensitive biomarker for detecting the presence of residual, or recurrent disease in patients with DTC (10). sTg, which is considered to be a more sensitive measure of tumor marker, can be measured either after the withdrawal of thyroxine or after the use of recombinant human TSH (rhTSH) injections. Both are comparable tests (6, 11). The value of sTg <1 ng/ml, represents a very low risk of recurrent/persistent disease (13). In some studies, specifically in low-risk patients, sTg has not played a major role (14–17). In contrast, other studies have concluded that Tg assay, during thyroid hormone suppression (suppressed Tg), might fail to detect thyroid cancer recurrence, residual tumor, or metastasis (18). sTg >2 µg/L identified all patients with metastases (19). Nevertheless, a single rhTSH-stimulated serum Tg <0.5 ng/ml in the absence of anti-Tg antibody has an approximately 98.0–99.5% likelihood of identifying patients that are completely free of the tumor at follow-up (20).

Recently, Tg measurement using high-sensitive assays have proven to be effective at reducing the need for Tg stimulation (11, 15). As confirmed by many studies using different high-sensitive assays, few patients with undetectable suppressed Tg have a pathological Tg response (>1 ng/ml) to stimulation (21, 22). They recommended that these patients need to be monitored through regular measurements of the suppressed Tg level and periodic neck ultrasonography. An increase in the levels of suppressed Tg should prompt further investigation (23). Further, suppressed Tg monitoring would prevent the dependence on the use of fixed cut-off criteria, as revealed in another study (24).

In this study we hypothesized that an undetectable Tg level in patients with DTC following total thyroidectomy and RIA ablation would ensure the absence of recurrence and avoid the use of stimulated Tg. It will also enable the establishment of a cut-off value for the suppressed Tg that will be used to decide whether further stimulation of thyroglobulin is needed.

## Materials and Methods

### Study Population

Prior to the recruitment, all the study procedures and protocols were reviewed and approved by the Institutional Review Board of the College of Medicine, King Saud University. Written informed consent was obtained from all the participants. We screened all the patients with confirmed DTC who were being followed-up at the thyroid cancer outpatient clinics at the King Khaled University Hospital in King Saud University Medical City, in Riyadh between February 2011 to February 2015. Their medical histories and physical examinations were recorded, and they underwent complete clinical radiological evaluation and laboratory investigations.

The inclusion criteria were: 1) age >14 and <75 years; 2) confirmed diagnosis of DTC based on the final pathological report with one or more of the following high-risk features indicate the treatment with RAI ablation, according to the available guidelines at that time (17, 25): indications for RAI ablation in our study patients was tumor size >2 cm, significant lymphovascular invasions (>3), gross extra-thyroidal extension, lymph node involvement (N1); 3) treatment with total thyroidectomy, followed by RAI ablation with iodine 131 (I-131). The decision for the dose was made by the thyroid cancer consultants based on a fixed dose approach, according to multiple variables including the pathology report, age of the patient, extension of the surgery, and the surgeon’s experience, and no pre-therapy scan was performed; and 4) non-stimulated or basal serum Tg (nsTg) level (<0.1) to <2 ng/ml, 3–6 months post RAI ablation.

Exclusion-criteria were: 1) age <14 years or >75 years and pregnancy; 2) those that did not require a complete surgery or RAI ablation because of the low risk disease; 3) patients who tested positive for Tg antibodies; 4) detectable persistent disease based on imaging, with positive uptake (outside the thyroid bed) in post ablation whole body scan (WBS) indicating metastasis, and/or if an ultrasound or computed tomography (CT) results showed significant remnant or suspicious lymph nodes or metastasis within 6 months of the diagnosis; and 5) patients who had highly suppressed Tg level (>2 ng/ml) 3–6 months post RAI ablation, since these indicate a high tumor marker that did not require further confirmation with Tg stimulation.

After 3–6 months following RAI ablation, the patients were stratified according to their nsTg or basal serum Tg (ng/ml) level into two groups: the first and second groups had Tg <0.1 and nsTg of 0.1–2.0 ng/ml, respectively. The serum Tg levels were measured while TSH was suppressed (0.1–0.5 µIU/ml). sTg was measured by rhTSH stimulation test protocol which required two intramuscular injections of rhTSH (thyrogen) 0.9 mg to be given on days 1 and 2. sTg was measured on day 5 (72 h after the second injection of thyrogen). The sTg test was considered to be negative when the level was <1 ng/ml and positive if >1 ng/ml. The first sTg was measured 3 to 6 months following the first RAI ablation while the second one was 5 years after the first one, for all patients.

Follow-up was performed until February 2020 every 3 to 12 months along with the laboratory investigations, including thyroid function test, and thyroglobulin and antithyroid antibodies, with a yearly neck ultrasound that was performed at the radiology department. Neck, chest, and abdominal CT scan, WBS using positron emission tomography (PET) scan, magnetic resonance imaging (MRI) of the brain, skeletal MRI, bone scan, and/or fine needle aspirations were performed for some patients with suspected structural disease.

At the end of the study in February 2020 (at least 5 years of follow-up for each patient), we classified the patients whose response to total thyroidectomy and RAI ablation concurred with ATA 2015 classification into excellent or indeterminate response, or biochemical or structural incomplete response groups (7). Excellent response was defined as negative imaging, and either nsTg <0.1 or sTg <1 ng/ml. The indeterminant response was defined as non-specific finding on imaging, faint uptake in the thyroid bed in RAI scan, nsTg 0.2–1.0, sTg 1.1–10.0, or stable anti-Tg antibodies or declining levels with the absence of structural or functional diseases. The biochemical incomplete response was defined as negative imaging and nsTg ≥1 or sTg ≥10 or rising anti-Tg antibody levels. The structural incomplete response was defined as structural or functional evidence of disease with any Tg level with or without anti-Tg antibodies.

### Laboratory Investigations

Laboratory investigations were conducted to measure serum Tg, TSH, FT4, and anti-thyroglobulin antibodies (ATg) levels. Serum concentration of Tg, TSH, and FT4 were measured by electrochemiluminescence immunoassay (ECLIA) system assay (Cobas, Roche Diagnostics, Germany). The detection limit of serum Tg was 0.04–500 ng/ml, and for TSH and FT4 assays, these were 0.005 µIU/ml and 0.023 ng/dl, respectively. The manufacturer’s reference range for TSH was 0.27–4.20 µIU/ml and for FT4 was 12–22 pmol/L (0.93–1.7 ng/dl). According to the manufacturer’s protocol, the reference ranges for all thyroid parameters were determined in 2003/2004 at the Universitätsklinikum Leipzig, Leipzig, Germany, from serum specimens collected from 870 blood donors. ATg were measured by enzyme-linked immunosorbent assay (ELISA) (INOVA Diagnostic, Ingbert, Germany), normal ranged from 0.0 to 0.6 units.

### Statistical Analysis

The IBM SPSS Statistics for Windows, version 20 (IBM Corp., Armonk, N.Y., USA) was used for the data analysis. Data are presented as mean, standard deviation, and percentage distribution. Independent t-test was used when the data were continuous and normally distributed. The chi-square test analysis was used for categorical data. The spearman’s correlation test was used to determine the association among non-parametric continuous variables.

## Results

Of 314 patients that were screened, 196 met our inclusion criteria. Subjects’ baseline demographics and clinical variables are summarized in **Table 1**. The majority of patients were females (n =161). The mean ( ± SD) age of the participants was 45 ( ± 12) years. The predominant histopathological finding for our patients was papillary thyroid cancer (n=175), with the following subtypes (classical n=112 64%, follicular variant n=49 28%, tall cell n=5 cell 3%, other variants n=9 5%) followed by follicular thyroid cancer (n=17), and Hurthle cell cancer (n=4). Indications for RAI were mainly lymph node involvement (N1a or N1b) in 109 patients (56%), T2 in 38 patients (19%), T3 in 52 patients (26.6%), and T4 in 14 patients (7%). Of the 196 patients, 13 (6.6%) had significant lymphovascular invasion while 3 had gross extra-thyroidal extension. More than half of the patients, 131/196 patients (67%), were treated with 3.7 GBq (100 mCi) RAI ablation; 41 patients (21%) were treated with 1.11 GBq (30 mCi), while 24 patients received 5.55 GBq (150 mCi).

**Table 1:** Baseline characteristics.

		Overall	Suppressed thyroglobulin (nsTg) ng/mL			
			Group 1 <0.1	>0.1–2		
				Group 2 >0.1–0.4	Group 3 0.5–1.0	Group 4 >1.0
**Patients n**		196	122	40	17	17
**Gender—n(%)**	**Male**	35 (17.9)	25(20.5)	5(12.5)	3 (17.6)	2 (11.8)
	**Female**	161 (82.1)	97(79.5)	35 (87.5)	14 (82.4)	15 (88.2)
**Age—years (SD)**		45 (12)				
**Pathology n (%)**	**PTC**	175	118	34	12	11
	**FTC**	17	3	6	3	5
	**HCC**	4	1	0	2	1
**sTg—n(%)**	**<1**	168 (85.7)	120 (98)		48 (65%)	
				34(85)	11(64.7)	3(17.6)
					26 (35%)	
	**=>1**	28 (14.3)	2 (2)	6(15)	6(35.3)	14(82.4)

Baseline characteristics of 196 patients following total thyroidectomy and radioactive iodine (RAI) ablations, with suppressed thyroglobulin below 2 ng/ml, were included from February 2011 to February 2015, with their non-stimulated and stimulated thyroglobulin measured 3–6 months following RAI ablation. PTC, papillary thyroid carcinoma; FTC, follicular thyroid carcinoma; HCC, Hurthle cell carcinoma; nsTg, non-stimulated thyroglobulin; sTg, stimulated thyroglobulin.

**Figure 1:**
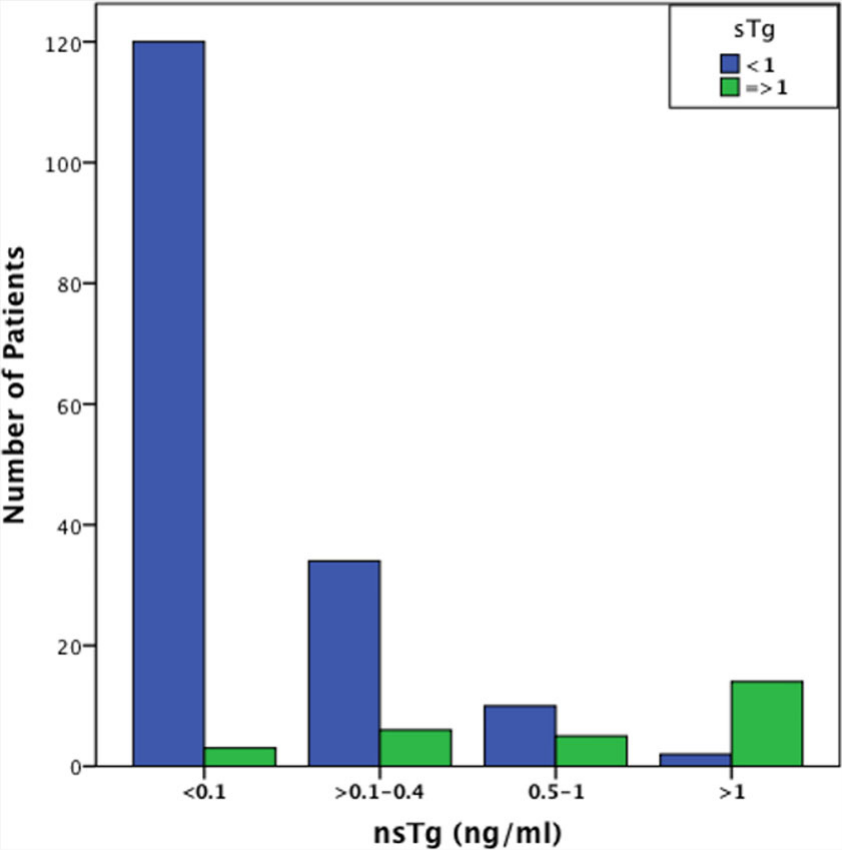
Graphical representation of the number of patients with sTg values < and >1 ng/ml in the different nsTg stratification groups. The graph demonstrate that most patients with low, undetectable nsTg <0.1 ng/ml also have low sTg levels. It also shows that an increase in the nsTg level leads to an increase in the sTg level that is significantly positively correlated (p <0.001). sTg, stimulated thyroglobulin; nsTg, serum non-stimulated thyroglobulin.

At 3–6 months post RAI therapy, the majority of the patients [n=122 (62.2%)] had basal suppressed serum or nsTg values of <0.1 ng/ml, in the majority of them [n=120 (98%)] also had sTg levels <1 ng/ml. Seventy four patients had nsTg values between 0.1 and 2.0 ng/ml and 26 of them (35%) had sTg >1 ng/ml (**Figure 1**). A strong correlation was found between the initial levels of nsTg and sTg at both the initial suppressed thyroglobulin (nsTg) <0.1 ng/ml and >0.1–2.0 ng/ml (r = 0.275, r = 0.821, p <0.01).

All the patients were followed-up from February 2011 until February 2020; median follow-up duration was 84 months.

### Patients With nsTg Level <0.1 ng/ml

**Figure 2:**
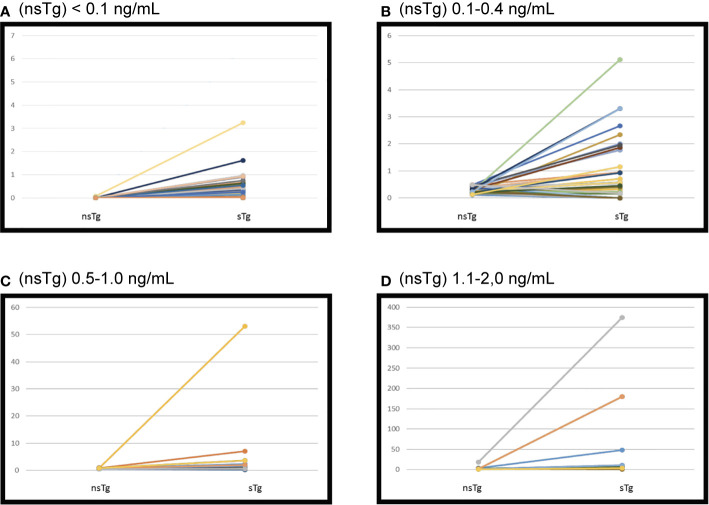
Initial suppressed thyroglobulin and stimulated thyroglobulin done 3–6 months following the radioactive iodine (RAI) ablation. Patients were grouped according to non-stimulated thyroglobulin. **(A)** Serum thyroglobulin levels before and after recombinant human TSH (rhTSH) stimulation in 122 patients had initial (nsTg) < 0.1 ng/ml****. sTg, stimulated thyroglobulin; nsTg, serum non-stimulated thyroglobulin. **(B)** Serum thyroglobulin levels before and after recombinant human TSH (rhTSH) stimulation in 40 patients had initial (nsTg) 0.1–0.4 ng/ml****. sTg, stimulated thyroglobulin; nsTg, serum non-stimulated thyroglobulin. **(C)** Serum thyroglobulin levels before and after recombinant human TSH (rhTSH) stimulation in 17 patients had initial (nsTg) 0.5–1.0 ng/ml****. sTg, stimulated thyroglobulin; nsTg, serum non-stimulated thyroglobulin. **(D)** Serum thyroglobulin levels before and after recombinant human TSH (rhTSH) stimulation in 17 patients had initial ****(nsTg) 1.1-2.0 ng/ml. sTg, stimulated thyroglobulin; nsTg, serum non-stimulated thyroglobulin.

Only 2 patients out of 122 (1.6%) had positive sTg (**Table 2** and **Figure 2A**). The first patient was a 32-year-old woman at diagnosis. Her initial pathology was 7 cm classical variant PTC with microscopic extra-thyroidal extension, positive surgical margins with >4 lymphovascular invasions, and 9 out of 33 lymph-nodes were positive at different levels of the neck, T4N1bM0. She received 5.55 GBq (150 mCi) RAI ablation initially. Her measured nsTg levels rose slowly during the 8-year follow-up to reach 3.6 ng/ml and the last sTg level was 11.2 ng/ml; however no structural disease was found. The second patient was 61-year-old man at diagnosis, with 7 cm encapsulated cystic PTC, a mixture of follicular and classical variant, and no extra thyroidal or lymphovascular invasion noted. The patient had only two negative lymph nodes resected (Nx). He received 3.7 GBq (100 mCi) RAI ablation, the nsTg level decreased over the 7-year follow-up period. The last sTg level was 1.4 ng/ml, and his last two nsTg levels were each <0.1 ng/ml, 6 months apart.

**Table 2:** Initial and Follow-up Stimulated Thyroglobulin.

		OVERALL	Initial suppressed thyroglobulin (nsTg) ng/mL			
			Group 1 <0.1	>0.1–2		
				Group 2 >0.1–0.4	Group 3 0.5–1.0	Group 4 1.0–2.0
**Patients n**		196	122	40	17	17
**Initial sTg n (%)**	**<1**	**168 (85.7)**	**120(98)**	**34(85)**	**11(64.7)**	**3(17.6)**
	**=>1**	**28 (14.3)**	**2 (2)**	**6(15)**	**6(35.3)**	**14(82.4)**
**Follow up sTg- n(%)**	**<1**	172(87.8)	121 (99)	35 (87.5)	**13(76)**	3(17.6)
	**=>1**	24(12.2)	1(1)	5(12.5)	4(24)	14(82.4)

Follow-up stimulated thyroglobulin results of the 196 patients following total thyroidectomy and radioactive iodine (RAI) ablation, with suppressed thyroglobulin <2.0 ng/mL. They were grouped according to the non-stimulated thyroglobulin levels 3–6 months following RAI ablation. Initial stimulated Tg (sTg) were measured 3–6 months following the RAI ablation. Follow-up sTg was measured after 5 years from the initial sTg measurement. nsTg, non-stimulated thyroglobulin; sTg, stimulated thyroglobulin.

**Figure 3:**
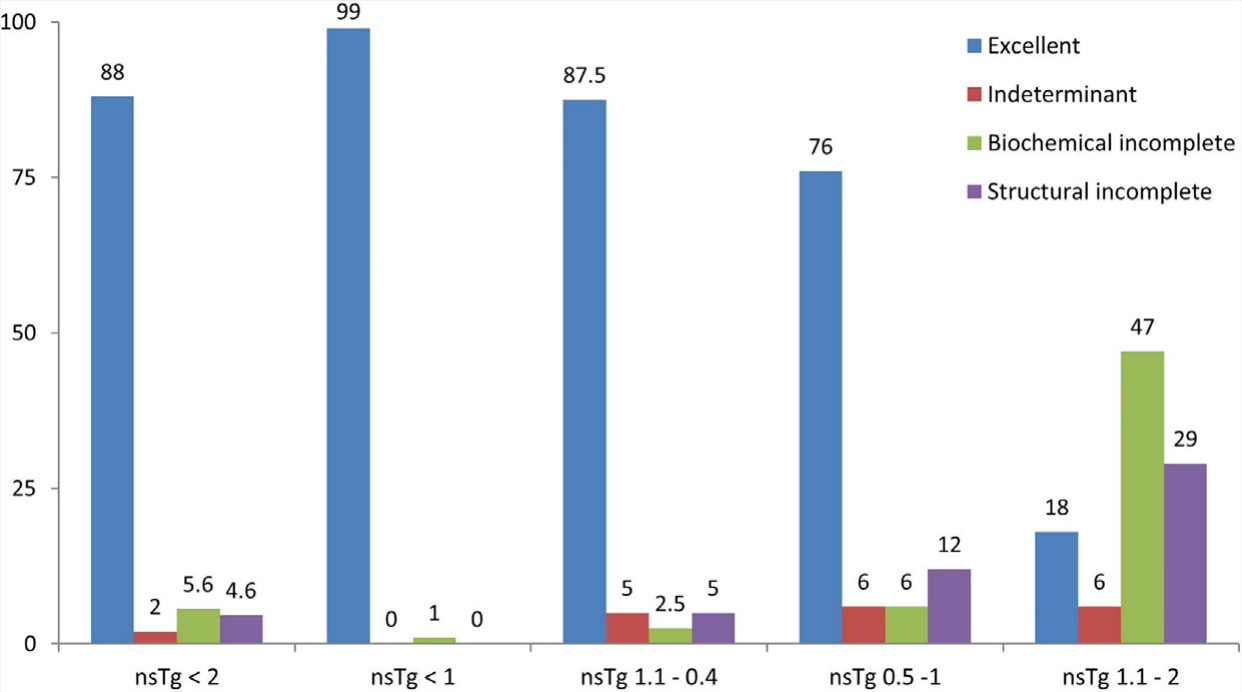
Graphical representation of the clinical response of patients in percentage (%) stated in February 2020, after a median follow-up of 84 months. 196 patients were included from February 2011 to February 2015 following total thyroidectomy and radioactive iodine (RAI) ablations, with initial suppressed thyroglobulin below 2 ng/ml done 3–6 months following the RAI ablation. Patients were grouped according to the initial non-stimulated thyroglobulin. nsTg, serum non-stimulated thyroglobulin.

The clinical responses of the patients were assessed (**Table 3**, and **Figure 3**) and we found that none of the patients had structural incomplete response, while one patient had biochemical incomplete response.

**Table 3:** Clinical response of the patients.

		Overall	Initial suppressed thyroglobulin (nsTg) ng/mL			
			Group 1 <0.1 ng/ml	>0.1–2 ng/ml		
				Group 2 >0.1–0.4 ng/ml	Group 3 0.5–1.0 ng/ml	Group 4 1.0–2.0 ng/ml
**Response n (%)**	**Excellent**	172(88%)	121(99%)	35(87.5%)	13(76%)	3(18%)
	**Indeterminant**	4(2%)	0	2(5%)	1(6%)	1(6%)
	**Biochemical incomplete**	11(5.6%)	1(<1%)	1(2.5%)	1(6%)	8(47%)
	**Structural incomplete**	9(4.6%)	0	2(5%)	2(12%)	5(29%)
Site of structural disease	Cervical	6	0	2	2	2
Mediastinum	2	0	1	1	1
Lung	3	0	0	0	3
Other	1	0	0	0	1

Clinical responses and the sites of the structural disease at the end of the study in February 2020, with median follow up of 84 months. 196 patients following total thyroidectomy and radioactive iodine (RAI) ablation were included from February 2011 to February 2015 with suppressed thyroglobulin <2.0 ng/mL. They were grouped according to the suppressed thyroglobulin levels 3–6 months following RAI ablation. nsTg, non-stimulated thyroglobulin.

Tg values 0.1–2.0 ng/ml occurred in 74 patients overall and 26 (35%) had positive sTg (>1 ng/ml). Among the 74 patients, nsTg levels between 0.1 and 0.4 ng/ml were found in 40 patients, and 34 (85%) of these had negative sTg. nsTg levels were between 0.5 and 1 ng/ml in 17 patients, 11 (65%) of whom had negative sTg. There were 17 patients with nsTg 1–2 ng/ml, and most of them [14 (82%)] had positive sTg >1 ng/ml.

### Patients With nsTg Level 0.1–0.4 ng/ml 

Overall, 34 out of 40 patients had sTg <1 ng/ml (**Table 2** and **Figure 2B**), and all of them had excellent response during the whole follow-up period (**Table 3** and **Figure 3**). The six (15%) patients had positive sTg two of them were diagnosed with structural incomplete response during the first year of the follow-up and both of them had cervical lymph-nodes and required surgery. One patient continued to have biochemical incomplete response. Two patients had indeterminant response. The last patient was reclassified to excellent response during the follow-up.

### Patients With nsTg Levels From 0.5 to 1.0 ng/ml

Out of 17 patients, 11 (65%) had negative sTg <1 ng/ml, and all had excellent response to the end of the follow-up (**Table 2** and **Figure 2C**). Six patients out of 17 (35%) had positive sTg, one patient had cervical lymph-nodes metastasis required multiple surgeries (3 over a 6-year follow-up). One patient had mediastinal mass required multiple RAI ablation (two times) with a total dose of 5.55 GBq (150 mCi), one patient had biochemical incomplete response and one had an indeterminant response. Two patients were reclassified to had excellent response. Of 17 patients, 13 (76%) achieved excellent response by the end of the follow-up (**Table 3** and **Figure 3**).

### Patients With nsTg level 1.1–2.0 ng/ml

Out of 17 patients, 14 (82%) had sTg >1 ng/ml (**Table 2**, and **Figure 2D**); 8 patients continued to have biochemical incomplete response, 5 (29%) developed structural incomplete response, 1 of them developed lymph nodes metastasis in the cervical area, 1 patient developed lymph nodes metastasis in the cervical and mediastinal areas, 2 patients had lung metastasis, and 1 patient had metastasis in multiple sites (the lung, bone, and liver). The first two patients underwent surgical treatment while the other three patients received multiple RAI ablations; one of them was administered a tyrosine kinase inhibitor. Three patients with sTg <1 ng/ml were followed-up for 6, 8, and 9 years; none of them developed structural or biochemical incomplete response (**Table 3** and **Figure 3**).

## Discussion

DTC is known to recur after the initial treatment. Although the rate of recurrence is low, it necessitates long-term follow up (26). The focus of this study was to evaluate the utility of sTg with suppressed or nsTg <0.1–2.0 ng/ml in DTC patients who were followed-up for 7 ± 2 years following total thyroidectomy and RAI ablation, to detect disease recurrence.

In our study, we observed a low basal undetectable nsTg in 122 patients. The levels of sTg remained undetectable (<1 ng/ml) in 121 patients (99%) and almost all had an excellent response at the end of the follow-up, with only one having a biochemical incomplete response. This finding indicates that patients with DTC with nsTg <0.1 ng/ml have a very low risk of recurrence; hence, it would be adequate to monitor these patients with an annual nsTg along with a periodic neck ultrasound as increasing serum nsTg levels are early and reliable indicators of recurrent disease (27). The finding also suggests that sTg testing does not change the overall management of DTC and is not required in this group of patients. Our findings are in line with previous studies, which have also proposed the use of highly sensitive Tg assay in low risk patients (23, 28). Our findings are in line with those of two other studies, which showed that patients with DTC having serum nsTg levels <0.1 ng/ml, are at low risk of recurrence, and that stimulated testing neither changes the management nor is needed in this group of patients (12, 23).

Rosario *et al*. showed comparable data with the same number of patients, in a retrospective study (n=194). We reached the same conclusion that patients with DCT, with a suppressed serum Tg <0.1 ng/ml rarely have an rhTSH-stimulated Tg >1 ng/ml. The previous study recommended monitoring of such patients with a suppressed Tg level and periodic neck ultrasonography (23).

sTg levels in our patients with nsTg level between 0.1 and 2.0 ng/ml 3 to 6 months after RAI ablations was sufficient to reclassify the patients from indeterminant response and biochemical incomplete response groups to the excellent response in a large group of patients who were confirmed over the 7 ± 2 years of follow-up period. These findings were consistent with those in some previous studies; however, the classification and reclassification approach and timing of sTg measurement differ. In some of the studies, the patients were classified either post-operatively, before RAI ablation, at the time of ablation, and a few months post RAI ablation in few studies, including ours. Most of the studies used the new and the old ATA classification while few used other classifications like the American Joint Committee on Cancer (AJCC) and the Union for International Cancer Control (UICC) (26, 29–32). In our study, we used the new ATA classification to classify and reclassify the patients 3–6 months post RAI ablation, and we continued to reclassify them during the minimum 5-year follow-up.

During the last four decades, Tg has been measured using three different methodological approaches: radioimmunoassay (RIA), immunometric assays, and liquid chromatography-tandem mass spectrometry (LC-MS/MS) that was developed in 2008. These assays differ mainly in their functional sensitivity potentials and propensity for HAMA and Tg Ab interference (20, 24, 33). Second-generation assays have functional sensitivity of ≤0.10  μg/L. Increasingly, laboratories are replacing the first-generation tests with Tg 2G IMA measurements. This is because of their superior functional sensitivities (34–36). The availability of these methods with higher functional sensitivity allows the measurements of Tg with good reliability, makes the surveillance of recurrences in apparently disease-free treated patients possible (23).

## Conclusion

In low risk thyroid cancer patients highly sensitive nsTg measurement <0.1 ng/ml identifies patients with DTC who are at a very low risk of recurrence and who do not need an sTg test. The sTg was able to reclassify a large group of patients with nsTg level 0.1 to 2 ng/ml and predict their response during a median follow up of 7 years. These findings enhance the use of sTg in this group of patients with DTC as well as dynamic risk assessment.

## Data Availability Statement

The original contributions presented in the study are included in the article. Further inquiries can be directed to the corresponding author/s.

## Ethics Statement

The studies involving human participants were reviewed and approved by Health Sciences Colleges Research on Human Subjects King Saud University College of Medicine, IRB Approval of Research Project No. E-20-4877. The patients/participants provided their written informed consent to participate in this study.

## Author Contributions

All authors contributed to the article and approved the submitted version.

## Conflict of Interest

The authors declare that the research was conducted in the absence of any commercial or financial relationships that could be construed as a potential conflict of interest.
